# Development of 3-phase fault detection, protection, and automation application with the present of DG in AC power system using GOOSE protocol

**DOI:** 10.1016/j.heliyon.2024.e27482

**Published:** 2024-03-08

**Authors:** Zhongsheng Shi, Zumrat Druzhinin

**Affiliations:** a Xinxiang Vocational and Technical College, Xinxiang, Henan, 453006, China; b Tajik Technical University, Tajikistan; c College of Technical Engineering, The Islamic University, Najaf, Iraq

**Keywords:** Protection relay, GOOSE protocol, IEC-61850, 3-Phase fault detection

## Abstract

In this research work, a protection and automation solution is developed, encompassing SEL751 and SEL751A protection relays that communicate through the IEC-61850 Generic Object-Oriented Substation Event (GOOSE) protocol to deliver high-speed detection and clearance of a 3-phase (3P) fault. The study case is a standard IEEE 13-bus grid including the main system generator (G1), power lines, loads, distributed generation (DG), bus bars, and feeders, equipped with protection relays for detecting over-current faults based on time and current. Two protection relays, B_1_ in the main system and B_2_ on the DG side, are integrated with a GOOSE protocol communication system. These settings are configured in such a way that, in the event of the main system breaker being disconnected, the DG cannot be connected to the network, making it a suitable setting for anti-islanding mode (AIM). The efficiency of the relay settings is tested by subjecting the power grid to a 3P fault, by the selected Time-Overcurrent (TOC) U3 inverse curve. Throughout the paper, the descriptions of the study, the grid, assumptions, settings calculations, and analysis of results are systematically presented. For the verification of relay settings, the performance of relays is practically tested and accurately analyzed in detail. The results obtained indicate that the presented strategy is quite effective for the configuration, setting operation, and coordination of relays for fast detection and communication through the GOOSE protocol.

## 1 Introduction

### 1.1 Background

To improve the performance of the power grid, various methods are presented by researchers every day to increase efficiency, optimization, reliability, resilience and network management [1,2]. In power systems, a protection relay plays a crucial role in safeguarding electrical equipment and ensuring the reliability of the power supply. A protection relay is an advanced device designed to monitor electrical parameters within a power system and initiate protective actions in the event of abnormal conditions or faults. Its primary purpose is to detect and isolate faults swiftly to prevent damage to equipment, minimize downtime, and maintain the overall integrity of the power system [3]. Overall, the key functions and characteristics of the protection relays in power systems can be classified as following items [4,5]:

• **Fault Detection**: Protection relays continuously monitor parameters such as voltage, current, frequency, and phase angles. They are capable of identifying abnormal conditions, including short circuits, overloads, and other faults.
• **Quick Response**: In the event of a detected fault, the protection relay must act swiftly to isolate the affected part of the system. Rapid response helps prevent damage to equipment and minimizes the impact on the rest of the network.
• **Selective Tripping**: Protection relays are designed to selectively trip specific circuit breakers or disconnect specific components to isolate the faulted area while keeping the rest of the system operational.
• **Communication**: Modern protection relays often feature communication capabilities, enabling them to exchange information with other relays or control systems. This facilitates coordinated protection schemes and enhances overall system reliability.
• **Diverse Protection Functions**: Protection relays can be configured for various protection functions, such as overcurrent (OC) protection, distance protection, differential protection, and others, depending on the specific requirements of the high-voltage system.
• **Adaptability**: Protection relays are adaptable to different voltage levels and types of electrical systems, making them versatile components in power distribution and transmission networks.
• **Monitoring and Recording**: Some protection relays are equipped with monitoring and recording capabilities, allowing operators to analyze system events, track the performance of protective devices, and optimize system reliability.

Breakers or automatic switches fitted with protection relays are extensively employed to safeguard electrical circuits from the risks of overload or short circuits, a common practice within the power grid. These devices require safeguarding due to the potential for significant, irreparable damage and the risk of fire in the event of a fault occurring on a high-voltage line. Consequently, the necessity for protection becomes paramount [[6], [7]]. Relay protection serves as an indispensable component of power systems, tasked with the crucial responsibility of identifying and isolating faults to prevent harm to equipment and ensure the stability of the system [8]. Traditional approaches to protection rely on the measurement of current and voltage to identify faults and initiate appropriate responses, such as tripping circuit breakers. These approaches predominantly hinge on extensive hardwiring between protection relays and associated devices [9]. Various strategies for designing protection relays in power systems exist. For example, in a paper by Ref. [10], the authors investigated Hardware-in-the-Loop (HIL) testing for SEL-351 protective relays in a sample distribution system employing Real-Time Digital Simulator (RTDS). The study delves into relay settings calculations, configuration procedures, and fault analyses, serving as a valuable resource for researchers engaged in HIL experimentation and enhancing comprehension applicable to intricate power systems.

Each protective system integrates several protection relays, with some types described herein. Fault detection techniques in overcurrent (O/C) relays encompass time-based, current-based, and current-time-based diagnoses. The overarching objective within protection is to delineate the healthy segments of the system from the faulty ones [11], with further classification of error detection methods provided in the subsequent paragraph.

Among the pivotal relays for transmission lines’ protection are O/C relays, tasked with identifying O/C conditions within the line and mitigating associated faults. O/C relays are typically classified based on error detection methodologies and operating times. In terms of fault detection, various relay types are typically incorporated [12,13], relays generally include the following types:

• Overcurrent
• Earth Fault (E/F)
• Directional Over Current
• Directional Earth Fault
• Sensitive Earth Fault
• Also, depending on the operating time, relays include the following types:
• Fixed Time Definite Time
• Inverse Time
• Instantaneous

In this paper, the implementation and performance analysis of a protection relay in a power network, comprising a transformer, bus bar, and feeder, for detecting O/C errors based on time and current, are desired. This method is elucidated.

### 1.2 Detection of O/C relays based on current/time

In this method, the relays are adjusted so that the cutoff time decreases as the error current increases, meaning that higher currents trigger quicker operating times. These relays are known as Inverse Definite Minimum Time (IDMT) relays [14]. IDMT relays operate based on predefined inverse curves, including: 1- Standard Inverse (SI), 2- Very Inverse (VI), 3- Extremely Inverse (EI), 4- Definite Time (DT).

**Fig. 1:**
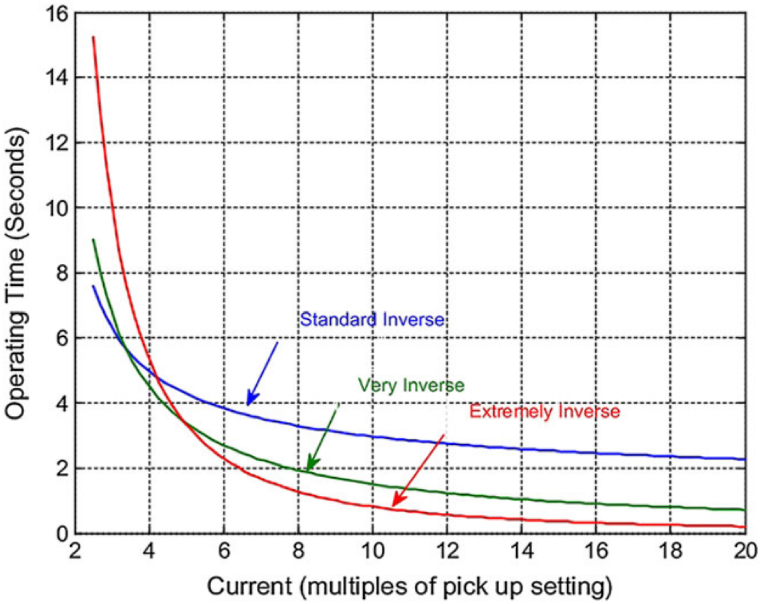
Inverse time-O/C curves [17].

Fig. 1 illustrates the inverse time-O/C performance curves of these relays. Additionally, O/C relays can detect ground faults. To ensure proper operation, the relay's input currents must align with the specified adjustment of the secondary current of the CT or current transformer. If the relay's input current is three-element, it can detect O/C errors; if it's four-element, it is able to detect ground errors [15]. For example, the ANSI code standard defines 51/50 for O/C (overcurrent) and 50 N/51 N for E/F (earth fault). The codes 50 and 50 N signify instantaneous operation of current relays, while 51 and 51 N denote delayed operation. During the inverse time delay, higher currents lead to shorter trip times, while lower currents lead to longer trip times. These relays receive input from the CT that is able to sample the current from the main line as well as transmitting it to the relay for analysis and operation [16].

In a power network, the protection of different parts of the network must be done through relays that are well coordinated for timely error detection and isolation. The basic rule in relay coordination is to first use identical relays in a series protection arrangement, as well as to ensure that the farthest relay from the power supply has a higher time setting than the upstream relay (relays close to the power supply).

### 1.3 Introduction to GOOSE protocol

The GOOSE protocol is an essential component of the International Electrotechnical Commission's (IEC) 61850 standard for substation automation. It facilitates high-speed communication and enables the exchange of data between intelligent electronic devices (IEDs) in a substation. GOOSE messages, transmitted over Ethernet networks, offer significant advantages over traditional hardwiring, including faster response times, reduced wiring complexity, and increased flexibility in reconfiguring protection schemes [16].

The GOOSE protocol operates based on a publisher-subscriber model, where an IED acting as a publisher sends GOOSE messages containing event data to multiple subscribers (other IEDs or relays) within the substation. These messages are multicast and provide real-time updates on system conditions, enabling synchronized actions among devices [18].

### 1.4 Integration of DG in AC power systems

DG systems, such as wind turbines and solar photovoltaic installations, are being increasingly integrated into AC power systems to enhance sustainability and support decentralized generation [19,20]. However, the presence of DG introduces new challenges to traditional protection schemes due to the bidirectional power flow, intermittent nature of renewable energy sources, and potential fault current contributions from DG during faults [21,22].

To address these challenges, advanced fault detection, protection, and automation techniques need to be developed. The proposed application aims to detect and mitigate 3-phase-to-ground faults in the presence of DG by utilizing the GOOSE protocol for fast and coordinated protection actions [23,24].

### 1.5 Motivation and main contributions of the paper

• Rising Importance of Relay Protection: Given the critical role of relay protection systems in maintaining the integrity of electrical power systems, the paper is motivated by the growing significance of developing advanced techniques to safeguard against faults.
• Challenges Posed by DG: The integration of DG in AC power systems introduces dynamic and complex challenges. The paper is motivated to address these challenges by enhancing fault detection, protection, and automation methods.
• Focus on 3-Phase Fault Detection: The main contribution of the paper lies in its focus on the development of a 3-phase fault detection, protection, and automation application. This targeted approach aims to address a specific aspect of system protection crucial for reliable operation.
• Utilization of GOOSE Message Protocol: The paper contributes to the field by utilizing the GOOSE message protocol for communication. This choice emphasizes the need for effective data exchange and coordination among relays and IEDs.
• Validation through Comparison: The paper validates its proposed methodology by comparing calculated results with simulation measurements. This contributes to establishing the reliability and accuracy of the developed application in real-world scenarios.
• Demonstrated Performance and Timely Response: The results showcase the application's performance, demonstrating that appropriate relay settings yield proportional and timely responses.
• Seamless Data Exchange with GOOSE Protocol: The successful and flawless performance of sending and receiving data through the GOOSE protocol is highlighted as a key contribution. The absence of delays underscores the protocol's efficiency in network protection.
• Efficient Network Protection through Accurate Settings: The overall contribution of the paper lies in proposing a protection strategy utilizing the GOOSE protocol, which efficiently provides network protection through precise settings and calculations.

In summary, the paper's motivation stems from the need to address challenges posed by DG in power systems. Its primary contribution lies in the development of specific 3-phase fault detection, protection, and automation applications, emphasizing the utilization of the GOOSE protocol for effective communication and coordination among relays and IEDs. The validation, demonstrated performance and efficiency of the proposed strategy further enhance its significance in the field of advanced power system protection.

### 1.6 Paper structure

The rest of this research work is categorized as follows: In Part 2, the methodology and implementation of relay protection are presented. Part 3 contains the calculations of settings for relay protection, while Part 4 illustrates the results and test verification of the case study, and then we will discuss about the obtained results in Part 5. Finally, the research paper is concluded in Part 6.

## 2 Methodology and implementation

### 2.1 Application of SEL-751(A) relay

The SEL 751(A) is classified as a versatile protective relay employed for safeguarding and managing power systems, including feeder, transformer, and motor protection. Offering a wide array of functions, it encompasses OC, under-voltage, over-voltage, and frequency protection, among others. This relay enhances control scheme flexibility by incorporating time and instantaneous OC, under-voltage, overvoltage, and frequency elements through a breaker fault protection system [25]. The utilization of the SEL-751A relay enables the design and configuration of SEL devices within IEC 61850 installations via the SEL QuickSet Software and Architect application. The Architect application facilitates the documentation and configuration of IEC 61850 communications between SEL devices and devices from various manufacturers.

Architect is instrumental in documenting and configuring IEC 61850 systems, encompassing GOOSE messages, Manufacturing Message Specification (MMS), and Sampled Values for process bus and Supervisory Control and Data Acquisition (SCADA) applications. The IEC 61850 GOOSE protocol offers an Ethernet-based solution for swift bus tripping that ensures interoperability with relays from compliant sellers [26,27].

### 2.2 Problem definition

**Fig. 2:**
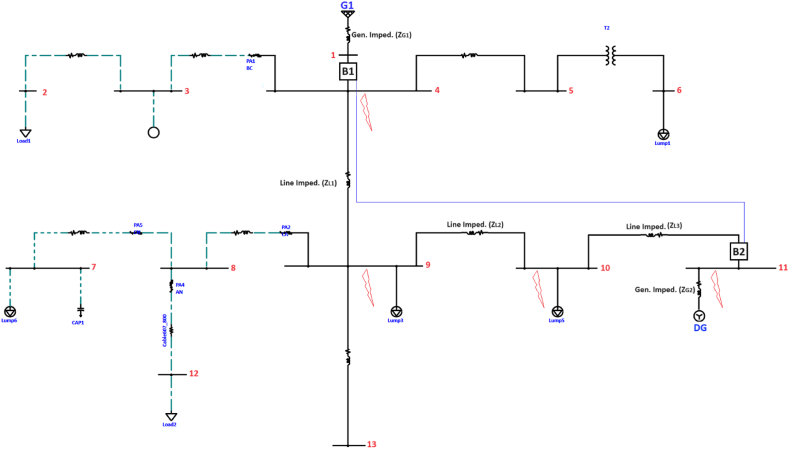
The assumed IEEE 13 bus power grid for the setting of a fast bus-tripping system.

Given that the setting, configuration, and coordination of relays are contingent upon the network they protect, an initial assumption is made, presuming the availability of a sample network for examination. Based on the functional requirements, including the need for a DG resource to be connected to the system and the presence of an auto-reclose system on the feeder, the IEEE 13 bus system is suitable for the application. The IEEE 13 bus system is commonly used for distribution system studies and includes a network configuration with 13 buses. It allows for the representation of distribution feeders and the interconnection of distributed generation resources. With the IEEE 13 bus system, we can model the main generator and distributed generation resources at appropriate bus locations to conduct relay settings and protection and automation application studies. Additionally, the presence of a feeder with an auto-reclose system aligns with your project requirements. Therefore, the IEEE 13 bus system provides a suitable network topology for designing and testing the protection and automation application required for interconnecting a distributed generation resource to a distribution feeder. Within this study, a main bus relay on the system side is utilized, establishing communication with the feeder relay on the DG side. Our assumed power grid for the configuration of a rapid bus-tripping system is depicted in Fig. 2. The locations of 3-phase fault to ground are given in this Figure.

G1 functions in bus 1 as the primary system generator, while G2 in bus 11 serves as the generator for the DG. The connection of DG to the system is contingent upon the feeder being linked to the grid system at the sourcing station. The feeder is equipped with an auto-reclose system, allowing it to reclose after a 3-s delay. This delay facilitates the restoration of service to other customers on the line if the disconnection of generation from the system at B_2_ is confirmed. If the fault persists after the first reclose, the breaker is opened and locked as a precautionary measure.

The primary system relay features a time-O/C curve to coordinate O/C with the DG relay. Additionally, it incorporates an instantaneous O/C element with a time delay to swiftly clear the bus during faults. When a fault occurs in the power grid, both relays detect the fault and transmit block signals to one another. Simultaneously, the relay transmits a GOOSE message, signaling to the other relay that a fault has transpired in the power grid. Subsequently, the relay inhibits the operation of the definite-time O/C element and continues timing on its time-O/C curve. At the time of occurring a fault, the main system relay transitions into its instantaneous curve after a specified time, ensuring it is not blocked by the DG relay. This unique feature decreases folding time, consequently mitigating the risk of arc flash and damaging equipment. In cases where only the main system relay identifies the fault, it promptly detects the issue. Since no message is received from the DG relay, the main system relay promptly initiates a trip, swiftly disconnecting the main breaker. This sophisticated coordination and communication between relays enhance the overall reliability and responsiveness of the protective system, minimizing potential damage and ensuring a swift and efficient response to faults in the power grid.

### 2.3 Assumption

The project's functional requirements are outlined as follows.

• The connection of the DG to the system is permissible only if the feeder is linked to the grid system at the sourcing station.
• The feeder is equipped with an auto-reclose system, which initiates reclosure only after a 3-second delay. This delay allows for the restoration of service to other customers on the line if the disconnection of generation from the system at B_2_ is confirmed. If the fault persists after the first reclosure, the breaker will be opened and locked as a precautionary measure.
• Location B_1_ is equipped with SEL751 relay, while location B_2_ has SEL751A relay.

The network values for setting both protection relays with instantaneous and inverse-time protective functions are specified as follows.

• All voltages are assumed to be 1.00 per unit (p.u.).
• The main generator impedance is *X*_SYS_ = 0.45 p.u. on bus 1.
• The DG impedance is *X*_DG_ = 0.7 p.u. on bus 11, and the apparent power of DG is *S*_DG_ = 3 MVA.
• The apparent power of load 1 on bus 9 is *S*_L1_ = 1 MW + 0.9 MVAR, and that of load 2 on bus 10 is *S*_L2_ = 1 MW + 0.7 MVAR.
• The base power and voltage values of the systems are set to 100 MVA and 25 kV, respectively. The system operates at 25 kV.
• All sequence impedances are equal, with positive sequence impedance for each device.

Each relay will be equipped with a definite time O/C protective function and an inverse-time O/C function to safeguard the entire length of the line. The base power of the system can be denoted as S_B_ = 1.0 (p.u.). Based on the system's assumptions, the base values of apparent power, voltage, current, and impedance are given in Table 1. Furthermore, it is necessary to acquire the impedances of both generators and all three segments of the line in Table 2. In order to calculate these values, there are equations related to them in Refs. [28,29].

**Table 1:** Base values.

Type	Value
$S_{B}$	$100\times 10^{6}\left(V.A\right)$
$V_{B}$	$25\times 10^{3}\left(V\right)$
$I_{B}$	$2.309\times 10^{3}\left(A\right)$
$Z_{B}$	6.25(Ω)

**Table 2:** Impedances of the generators and lines.

Type	Value
$Z_{G1}$	2.813i(Ω)
$Z_{G2}$	4.375(Ω)
$Z_{L1}$	0.875i(Ω)
$Z_{L2}$	1.5i(Ω)
$Z_{L3}$	0.5i(Ω)

Where Z_G1_ and Z_G2_ represent the impedances of the main generator on bus 1 and DG on bus 11. Z_L1_, Z_L2_, and Z_L3_ are lines impedances. To assess the performance of the relay settings and in alignment with the chosen TOC-U3 inverse curve, the system's ability to detect and clear a three-phase-to-ground fault close to the load side is tested. Additionally, one of the objectives of this research is to investigate the influence of the IEC 61850 GOOSE message and communication link. To establish appropriate settings in the relays, the values of the following elements must be determined: 51P1P (time O/C trip pickup), 51P1T (time O/C trip), VB001 (block. signal), 50P1P (max. phase O/C trip pickup), and 50P1T (max. phase O/C torque).

## 3 Strategy and calculations

In order to ensure rapid clearing of three-phase faults and design and implement a protection and automation strategy, it is essential to establish the base values. It is required to have loads power in the complex format, as well as the impedances of loads, which are obtained from equations (1), (2), (3), (4).

(1) $$S_{L1}=\left(1-0.9i\right)\times 10^{6}\left(VA\right)$$

(2) $$S_{L2}=\left(1-0.7i\right)\times 10^{6}\left(VA\right)$$

(3) $$Z_{P1}=\frac{{V_{B}}^{2}}{S_{L1}}=\left(345.304+310.773i\right)\left(\Omega \right)$$

(4) $$Z_{P2}=\frac{{V_{B}}^{2}}{S_{L2}}=\left(419.463+293.624i\right)\left(\Omega \right)$$

The next step is to calculate the max. fault current of G1. The current seen by B_1_ using equation (5) is calculated.

(5) $$I_{F\_P1\_G1}=\frac{V_{B}}{Z_{G1}+Z_{L1}}=\left(6.78\times 10^{3}∠-90{}^{\circ}\right)\left(A\right)$$

Where $I_{F\_P1\_G1}$ represents the fault current of the line supplied by G_1_. This amount needs to operate at least after 0.4 s. Additionally, the fault at DG observed by the B_1_ relay should be calculated. To determine the current in this electrical circuit, obtaining the voltage with equivalent impedances in series and parallel forms is necessary. Following this, we will have equation (6):

(6) $$I_{F\_G2\_G1}=\frac{V_{B}}{Z_{G1}+Z_{L1}+\frac{\left(\frac{\left(Z_{G2}+Z_{L3}\right)\times Z_{P2}}{\left(Z_{G2}+Z_{L3}\right)+Z_{P2}}+Z_{L2}\right)\times Z_{P1}}{\left(\frac{\left(Z_{G2}+Z_{L3}\right)\times Z_{P2}}{\left(Z_{G2}+Z_{L3}\right)+Z_{P2}}+Z_{L2}\right)+Z_{P1}}}=\left(3.506\times 10^{3}∠-52.997{}^{\circ})(A\right)$$

Where $I_{F\_G2\_G1}$ represents the fault current seen by G_1_ when the fault is in front of G_2_. It is assumed that all loads are supplied from one end. It is essential to ensure that there is no tripping on load, so calculating the largest served loads is necessary. Therefore, the amount of power and current that should be provided by the main system generator G_1_ is obtained from equations (7), (8) in succession.

(7) $$S_{G1\_load}=S_{L1}+S_{L2}=\frac{V_{B}}{Z_{G1}+Z_{L1}}=\left(2.5611\times 10^{6}∠-38.66{}^{\circ}\right)\left(VA\right)$$

(8) $$I_{G1\_load}=\frac{S_{G1\_load}}{\sqrt{3}\times V_{B}}=\left(59.15∠-38.66{}^{\circ}\right)\left(A\right)$$

The next step is to select the circuit transformer ratio (CTR). Usually, there are some standard CTRs to be used such as 20, 50, 100, 150, 200, 250, 300, 600, 800, 1000, and 1200 [30]. The nominal value of the CT secondary current must be below 1 A for normal load conditions and up to 20 A for fault conditions. A CTR value of 1000 is chosen for calculation purposes. With the selected CTR, the current supplied by the main system generator G1 of CT can be calculated using Eq. (9).

(9) $$I_{G1\_load\_CT}=\frac{I_{G1\_load}}{CTR}=\left(0.059∠-38.66{}^{\circ}\right)\left(A\right)$$

Next, we need to calculate the pickup current of G_1_ measured by CT. Based on the setting, it can be between 2 and 3 times bigger than the current load supplied by the main system generator G_1_. Based on the U3 (very inverse) curve given by the instruction manual of the relay, it should be in an acceptable range. Therefore, equation (10) can be used:

(10) $$I_{pickup\_G1}=2.5\times I_{G1\_load\_CT}=\left(0.148∠-38.66{}^{\circ}\right)\left(A\right)$$

Considering the CTR, the current seen by B_1_ in CT using equation (11).

(11) $$I_{F\_P1\_G1\_CT}=\frac{I_{F\_P1\_G1}}{CTR}=\left(6.78∠-90{}^{\circ}\right)\left(A\right)$$

The multiple of the pickup value (M) needs to be obtained for G_1_. Based on the U3 curve given by the instruction manual of the relay, it should be in an acceptable range. Therefore, the maximum amount of M can be calculated using equation (12):

(12) $$M_{\max}=\frac{\left|I_{F\_P1\_G1\_CT}\right|}{\left|I_{pickup\_G1}\right|}=45.848$$

As given earlier, the tripping time should be around 0.4 s ($t_{p1}=0.4$). The Time Dial (TD) is set based on the next equation, which is the lowest TD value available for special protection of the grid. Therefore, equation (13) is given.

(13) $$TD=\frac{t_{p1}}{\left(0.0963+\frac{3.88}{{M_{\max}}^{2}}\right)}=4.076$$

Similarly, for fault at DG (G_2_) point, the tripping current at the secondary of the CT is given by equation (14):

(14) $$I_{F\_G2\_G1\_CT}=\frac{I_{F\_G2\_G1}}{CTR}=\left(3.506∠-52.9971{}^{\circ}\right)\left(A\right)$$

Here, the amount of M should be calculated for G_2_. The minimum amount of M can be obtained from equation (15):

(15) $$M_{\min}=\frac{\left|I_{F\_G2\_G1\_CT}\right|}{\left|I_{pickup\_G1}\right|}=23.709$$

The tripping time at G_2_ as seen from G_1_ can be given as [equation (16)]:

(16) $$t_{p2}=TD\times \left(0.0963+\frac{3.88}{{M_{\min}}^{2}-1}\right)=0.421$$

As can be seen, the difference between the two obtained amounts of tripping time (t_p1_-t_p2_) is 21 milliseconds which shows how fast the rely trips at the faulty G_2_ location versus the G_1_ location which is a good number. To set the definite time O/C for fault at location G_1_, the calculation of the current fault using equation (17) needs to be performed.

(17) $$I_{F\_G1}=\frac{V_{B}}{Z_{G1}}=\left(8.889\times 10^{3}∠-90{}^{\circ}\right)\left(A\right)$$

This current in the secondary of the CT will be [equation (18)]:

(18) $$I_{F\_G1\_CT}=\frac{I_{F\_G1}}{CTR}=\left(8.889∠-90{}^{\circ}\right)\left(A\right)$$

It is assumed that the setting needs a 10% coefficient, so the current for element 50 of the relay works with no time delay using equation (19):

(19) $$I_{50\_set}=\left|I_{F\_P1\_G1\_CT}\times 1.1\right|=7.458\left(A\right)$$

The obtained values for elements 51 and 50 settings are given in Table 3, Table 4.

**Table 3:** Obtained values for element 51 setting.

Type	Value
CTR	1000
TD	4.076
I pickup	0.148
Curve	U3

**Table 4:** Obtained values for element 50 setting.

Type	Value
I_TRIP	7.458
Tdelay	0.0
Curve	U3

## 4 Results

**Fig. 3:**
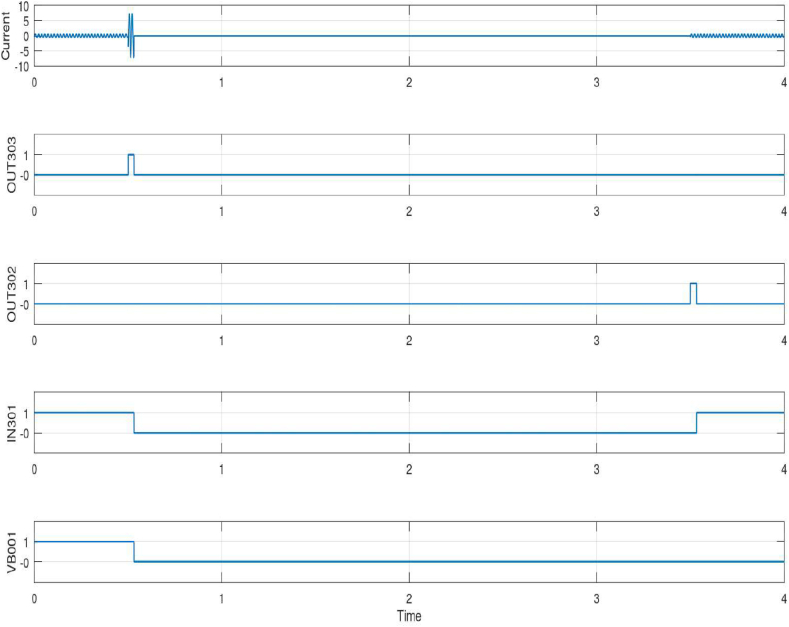
The result of the input and output signals of the B_1_ relay.

The next step involves selecting testing values for the protecting relay operating characteristics. To evaluate the efficiency of the relay settings, tests are conducted on relays in various states, in accordance with the selected U3 inverse curve. After loading the relay settings and making sure the connection of two relays is correct and determining the test modes, we test the relays in the laboratory. The time to complete the whole test is 4 s, the 3P fault occurs in the first 0.5 s. After 3 s and the line is cleared, the relay connects once to the main network breaker and the DG breaker remains in disconnected (open) mode. Fig. 3 shows the result of the input and output signals of the B_1_ relay. It should be noted that the fault occurs in 0.5 s and is cleared in 3.5 s and the grid is re-energized, so the total time of the fault and power outage is 3 s. This information is displayed in the Human Machine Interface (HMI) after processing. HMI is used to monitor, view, and adjust the parameters of industrial devices such as Programmable Logic Controller (PLC) and inverters. HMI is a monitor that can be programmed and with its help, we can change various parameters and control the system.

As shown in Fig. 3 in the current section, the current curve has normal operation for the first half second and there are no faults. In 0.5 s, when the 3P trip occurs, and the short-circuit current passes several times the nominal current, according to the pickup current (I-_Pickup_) that is set in the relay settings, the B_1_ relay detects the fault and sends an OUT303 disconnection signal to the main system breaker. This signal is also sent to the other relay B_2_ through a GOOSE message, which shows that the main breaker of the grid has been disconnected (open).

Based on these results, it is shown that no signal from OUT302 is sent or displayed because this element sends and displays the close or reclose signal of the breaker. Hence, there is no reclosing of the circuit breaker in 0.5. Element IN301 is an indicator and receiver of the voltage of the main breaker, so it shows the close or open status of the main system breaker. After the inter-tripped of the breaker, the voltage signal value changed from 1 to 0 zero in 0.5 s.

As can be seen from VB001 (blocking the signal), in the first 0.5 s, this element receives the communication signal that indicates the close state of the DG breaker from relay B_2_ through the communication protocol and GOOSE message. After the trip, by opening the DG breaker, the blocking signal is received from the breaker and changes from 1 to 0, which indicates the open status of the DG breaker. Table 5 shows the results values of different elements for the B_1_ relay.

**Table 5:** The results values of different elements for the B_1_ relay.

B_1_ SER Records Date/TIME	ITEM	STATE
2024-02-16 13:35:42:180	50P1P	Assert.
2024-02-16 13:35:42:180	50P1T	Assert.
2024-02-16 13:35:42: 180	51P1P	Assert.
2024-02-16 13:35:42: 180	OUT303	Assert.
2024-02-16 13:35:42: 191	IN301	Deassert.
2024-02-16 13:35:42:191	SV01	Deassert.
2024-02-16 13:35:42:195	OUT303	Deassert.
2024-02-16 13:35:42:195	VB001	Deassert.
2024-02-16 13:35:45:181	OUT302	Assert.
2024-02-16 13:35:45:190	SV01	Assert.
2024-02-16 13:35:45:190	IN301	Assert.
2024-02-16 13:35:45:202	OUT302	Deassert.

As can be seen from Table 5, 50P1P (max. phase O/C trip pickup), 50P1T (max. phase O/C torque), and 51P1P (time O/C trip pickup) are asserted at 13:35:42:180. At the same time, element OUT303 asserted and sent an open signal. IN301 receives the open signal from the main breaker with a 11-ms delay, which confirms the correct operation of the relay, and breaker. After a 15-ms delay, elements OUT303 and VB001 are de-asserted which means the blocking signal is received from other relay B_2_.

After clearing the fault after 3 s, in 13:35:45:181, element OUT302 asserted which means the reclosing signal was sent to the main system breaker with a 1-ms delay. In addition, element IN301 asserted which shows the reclosing signal is sent to the main breaker with a 10-ms delay. Element OUT302 is de-asserted after 22 milliseconds that the main breaker is reclosed and the grid is powered. Similarly, there are results of the test for relay B_2_.

**Fig. 4:**
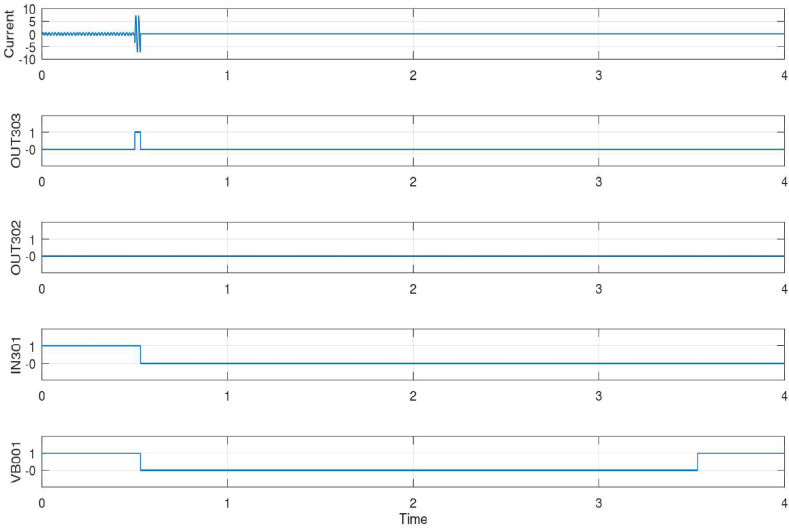
The result of the input and output signals of the B_2_ relay.

Fig. 4 shows the result of the input and output signals of the B_2_ relay. It should be noted that the fault occurs in 0.5 s and is cleared in 3.5 s and the grid is re-energized, so the total time of the fault and power outage is 3 s.

As shown in Fig. 4 in the current section, the current curve has normal operation for the first half second and there are no faults. When the fault signal is received by B_2_ from the main system relay B_1_, element OUT303 immediately sends an open signal to the DG breaker. In addition, the breaker open signal of DG is sent to the main relay B_1_, which indicates that the DG breaker is disconnected. This situation will be similar to when a fault has occurred separately in front of the B_2_ relay, and the B_2_ relay itself sees the fault. After 3 s and clearing the fault in the grid, even though the main breaker of the grid is closed, because the possibility of reclosing the DG breaker is not defined in the logic, the DG breaker will not be closed. As a result, OUT302 has not sent any breaker close signal. Also, element IN301 after opening and inter-tripping the breaker, by changing the signal from 1 to 0, shows that it received the trip signal and was opened in 0.5 s and remained disconnected until the end of the test. The VB001 element receives the communication and status of the corresponding relay B_1_. From the beginning of the test to the time of the fault in 0.5 s, it receives the connection status of the main system breaker from B_1_ and the power of the grid through the GOOSE message. From 0.5 to 3.5 s, when the main system breaker is opened due to the fault, B_2_ receives a 0 signal from the B_1_ relay. After clearing the fault and reclosing the main system breaker in 3.5 s, it receives the closing signal again from relay B_1_. Table 6 shows the results values of different elements for the B_2_ relay.

**Table 6:** The results values of different elements for the B_2_ relay.

B_2_ SER Records Date/TIME	ITEM	STATE
2024-02-16 13:35:42:181	50P1P	Assert.
2024-02-16 13:35:42:181	51P1P	Assert.
2024-02-16 13:35:42:181	50P1T	Assert.
2024-02-16 13:35:42:181	OUT303	Assert.
2024-02-16 13:35:42:184	SV01	Assert.
2024-02-16 13:35:42:185	VB001	Deassert.
2024-02-16 13:35:42:196	IN301	Deassert.
2024-02-16 13:35:42:201	OUT303	Deassert.
2024-02-16 13:35:45:201	VB001	Assert.

As shown in Tables 6 and in 13:35:42:181, elements 50P1P (max. phase O/C trip pickup), 50P1T (max. phase O/C torque), and 51P1P (time O/C trip pickup), and OUT303 of B_2_ is asserted, which indicates the fast operation of the relay to receive the opening and blocking signal. At this time the signal for opening the breaker of the DG is sent with 0.0 s delay. Also, element VB001 (block. signal) is de-asserted with a 3-ms delay, and after 15 milliseconds, element IN301 is de-asserted; also, element OUT303 is de-asserted after 20-ms delay which illustrates that the DG breaker signal is opened and has no voltage.

With clearing fault after 3 s, in 13:35:45:200, with a 20-ms delay VB001 is asserted, which confirms the correct operation of the relay to receive communication signal through GOOSE message. This shows that the main system breaker is reclosed and working in normal condition. However, the DG breaker remains open. In order to illustrate the advantages of the proposed protection relay approach, Table 7 provides a comparison of key aspects in relay testing vs. two simulation-based studies (references [31,32]).

**Table 7:** A comparison of key aspects in relay testing vs. simulation-based studies.

Criteria	Reference [31]	Reference [32]	Advantages of the proposed approach in this study
**Experimental Setup**	Simulation environment with symmetrical sources and distance protection relay implementation.	Microgrid system modeling using ETAP PowerStation with fault analysis and protection logic configurations.	Detailed relay testing in a laboratory setting simulating grid states.
**Fault Testing**	Simulation-based fault scenarios and validation of distance protection relay model.	Fault analysis in both grid-connected and islanded modes, considering various fault parameters.	Rigorous relay testing with simulated faults and clear presentation of relay response.
**Equipment and Systems**	Symmetrical sources, distance protection relays, and real-time simulation in HYPERSIM.	Microgrid system with HCPV solar, wind power, microturbines, and PCS 100 energy storage. Protection logic configurations using Arcteq_F215 IEDs.	Focus on relay settings, connections, and HMI for monitoring.
**Communication and Control**	Implementation of IEC 61850 modules for substation wiring reduction.	Detection of operating modes using GOOSE messages and MMS, with detailed protection logic configurations.	Emphasis on communication between relays using GOOSE messages.
**Results Presentation**	Validation of distance protection relay model with fault scenarios and real-time simulation results.	Comprehensive fault analysis, operating mode detection, and protection logic configurations are presented in tables and figures.	Detailed relay operation timing and signal states are presented in tables and figures.
**Conclusion**	Emphasis on simulation-based distance relay validation and fault scenarios.	A comprehensive study covering fault analysis, operating modes, and protection logic configurations in a microgrid system.	Focused on relay performance and fault testing in a laboratory environment.

## 5 Discussion

The primary focus of this paper revolves around creating and assessing a 3-phase fault detection, protection, and automation system that utilizes the GOOSE message protocol. The aim is to tackle challenges arising from the growing integration of DG in AC power systems and to ensure the dependable and efficient operation of relay protection systems. By comparing calculated results with laboratory measurements, valuable insights into the relay protection system's performance are gained. One significant observation is the close correlation between experimental and calculated values, especially concerning critical parameters like pickup and trip times. The successful de-assertion of element OUT302 after 22 milliseconds, corresponding to the main breaker's reclosure and the restoration of power to the grid, underscores the accuracy and reliability of the relay settings.

The study's findings validate the effectiveness of the GOOSE message protocol for data transmission, emphasizing its precision and seamless performance without noticeable delays. The precise relay data transfer achieved through the GOOSE protocol emerges as a crucial factor contributing to the efficiency of the protection strategy. This underscores the potential of the proposed approach in enhancing network protection in dynamic and complex electrical power systems.

The overall success of the relay protection system in the experimental setup, alongside the alignment between calculated and measured values, suggests that the developed application can offer robust fault detection and protection in the presence of distributed generation. The accurate settings and calculations, coupled with the reliable data transfer facilitated by the GOOSE protocol, enhance the system's overall reliability and effectiveness in ensuring the stability of AC power systems.

## 6 Conclusion

Relay protection systems are pivotal in ensuring the safety and reliability of electrical power systems by guarding against faults. As DG becomes increasingly integrated into AC power systems, it becomes essential to develop advanced fault detection, protection, and automation techniques capable of navigating these dynamic and intricate environments. This paper centers on crafting a 3-phase fault detection, protection, and automation application utilizing the GOOSE message protocol. The study employs the IEEE 13-bus system, which encompasses generators, power lines, loads, DG units, bus bars, and feeders, all outfitted with protection relays designed to detect overcurrent (OC) faults based on time and current parameters. The main generator, DG, and loads are distributed across different buses with varying line impedances. By comparing the calculated results with laboratory measurements, the study establishes a correlation between the experimental performance and the relay settings, indicating a close alignment between the two sets of data. Notably, element OUT302 de-asserted after 22 milliseconds, coinciding with the reclosure of the main breaker and the restoration of power to the grid. This demonstrates that the difference between the pickup and trip times in the calculations mirrors that observed in the testing phase. The performance of data transmission using the GOOSE protocol is found to be accurate and seamless, devoid of any discernible delays. The results underscore the efficacy of the presented protection strategy, facilitated by the GOOSE protocol's ability to transmit relay data accurately. Through precise settings and calculations, the protection strategy efficiently enhances network protection in AC power systems.

## Additional information

No additional information is available for this paper.

## CRediT authorship contribution statement

**Zhongsheng Shi:** Validation, Software, Resources, Methodology, Data curation, Conceptualization. **Zumrat Druzhinin:** Writing – review & editing, Writing – original draft, Software, Methodology, Formal analysis, Data curation, Conceptualization.

## Declaration of competing interest

The authors declare that they have no known competing financial interests or personal relationships that could have appeared to influence the work reported in this paper.
